# Supramolecular Nanorods of (N-Methylpyridyl) Porphyrin With Captisol: Effective Photosensitizer for Anti-bacterial and Anti-tumor Activities

**DOI:** 10.3389/fchem.2019.00452

**Published:** 2019-06-21

**Authors:** Raman Khurana, Aarti S. Kakatkar, Suchandra Chatterjee, Nilotpal Barooah, Amit Kunwar, Achikanath C. Bhasikuttan, Jyotirmayee Mohanty

**Affiliations:** ^1^Radiation & Photochemistry Division, Bhabha Atomic Research Centre, Mumbai, India; ^2^Homi Bhabha National Institute, Training School Complex, Mumbai, India; ^3^Food Technology Division, Bhabha Atomic Research Centre, Mumbai, India

**Keywords:** host-guest complex, captisol, porphyrin, singlet oxygen generation, antibacterial activity, antitumor activity

## Abstract

Porphyrins, especially the 5,10,15,20-tetrakis(4-*N*-methylpyridyl) porphyrin (TMPyP), are well-accepted as photosensitizers due to strong absorption from visible to near-infrared region, good singlet oxygen quantum yields as well as chemical versatility, all of which can be further modulated through planned supramolecular strategies. In this study, we report the construction of supramolecular nanorods of TMPyP dye/drug with captisol [sulfobutylether-β-cyclodextrin (SBE_7_βCD)] macrocycle through host-guest interaction. The availability of four cationic N-methylpyridyl groups favors multiple binding interaction with the captisol host, building an extended supramolecular assembly of captisol and TMPyP. In addition to the spectroscopic characterizations for the assembly formation, the same has been pictured in SEM and FM images as nanorods of ~10 μm in length or more. Complexation of TMPyP has brought out beneficial features over the uncomplexed TMPyP dye; enhanced singlet oxygen yield, improved photostability, and better photosensitizing effect, all supportive of efficient photodynamic therapy activity. The Captisol:TMPyP complex displayed enhanced antibacterial activity toward *E. coli* under white light irradiation as compared to TMPyP alone. Cell viability studies performed in lung carcinoma A549 cells with light irradiation documented increased cytotoxicity of the complex toward the cancer cells whereas reduced dark toxicity is observed toward normal CHO cells. All these synergistic effects of supramolecular nanorods of Captisol-TMPyP complex make the system an effective photosensitizer and a superior antibacterial and antitumor agent.

## Introduction

Photosensitizers play an important role in photodynamic therapy (PDT) where the photosensitizers have the ability of absorbing light energy and transfer that to surrounding oxygen to generate highly reactive singlet oxygen and thereby destroy the cancerous or diseased tissues or inhibit the microorganism growth (Lovell et al., 2010; Liu et al., 2013; Ormond and Freeman, 2013). Generally, the specific criteria for a good photosensitizer is that it should show strong absorption with a high extinction coefficient in the red/near infrared region of the electromagnetic spectrum (600–850 nm) and should have longer excited state lifetime, high photostability, high singlet oxygen quantum yield and low dark toxicity (Lovell et al., 2010). Porphyrins and their derivatives comprise of several properties, such as absorption in the wavelength range 350–800 nm, phototoxic upon light irradiation and singlet oxygen generation, low dark toxicity which render them preferential candidates as photosensitizers (Vermathen et al., 2013). However, the inherent self-assembling behavior of porphyrins in aqueous medium due to strong hydrophobic or π-π stacking interactions greatly affects/reduces the ability to generate singlet oxygen as the stacked molecules release the absorbed energy mainly as heat and thereby quench the fluorescence emission (Fernandez et al., 1996; Vermathen et al., 2013; Voskuhl et al., 2013). Covalent modification of porphyrins, involving time consuming tedious chemical synthesis and purification processes, is one of the ways to overcome this practical difficulty (Liu et al., 2013). Nevertheless, the non-covalent modification of porphyrins through macrocyclic hosts without affecting their chemical composition is another way to achieve the desired property of porphyrin for singlet oxygen generation and supramolecular photosensitizing behavior (Liu et al., 2013; Wang et al., 2016; Li et al., 2017, 2018; Rui et al., 2017; Semeraro et al., 2018; Gao et al., 2019). Among the huge collection of porphyrins, 5,10,15,20-tetrakis(4-*N*-methylpyridyl)porphyrin (TMPyP) has attracted a great deal of attention due to its efficient photosensitizing action in photodynamic therapy (Kaestner et al., 2003) and molecular assembly formation with macrocyclic receptors like cyclodextrins (Cosma et al., 2006), cucurbiturils (Mohanty et al., 2008), and calixarenes (Lang et al., 2001; Moschetto et al., 2002). In few recent studies, the enhanced antibacterial activity of TMPyP derivatives in the presence of cucurbit[7]uril against *E. coli* have been established (Liu et al., 2013; Chen et al., 2017). In an earlier study, we have shown the formation of stable and extendable supramolecular architecture of TMPyP with cucurbit[7]uril (CB7) having 1:4 stoichiometry (Mohanty et al., 2008). Furthermore, we have demonstrated the uptake and stimulus-responsive release of TMPyP from the cucurbituril-functionalized silver nanoparticle conjugates for the drug delivery application (Barooah et al., 2011). In another study, we have shown efficient interaction of TMPyP with single-strand DNA homopolymers, whereas, (dG)_40_ DNA significantly quenches the fluorescence intensity of porphyrin through photo-induced electron transfer from dG to TMPyP (Dutta Choudhury et al., 2014).

**Scheme 1:**
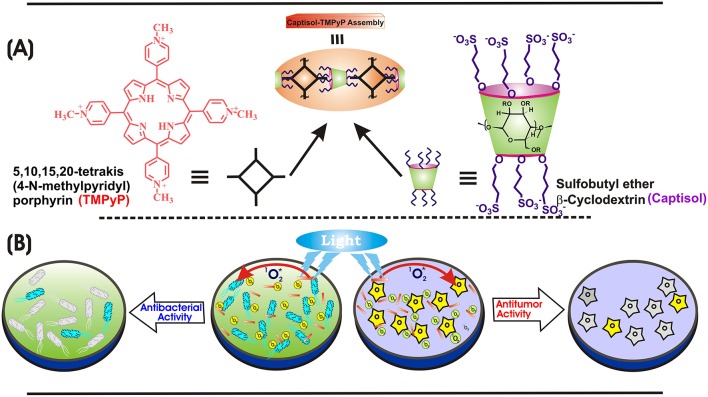
**(A)** Chemical structures of TMPyP, captisol and the representation of the Captisol:TMPyP complex. **(B)** Pictorial representation of the singlet oxygen generation from the TMPyP complex and killing of *E. coli* bacteria and *A549* cancerous cell lines.

In the recent past, several attempts have been made to functionalize the host and/or the guest molecules to control and tune the host guest interactions for targeted applications (Liang et al., 2012). In this context, captisol (SBE_7_βCD, Scheme 1), a chemically modified cyclodextrin moiety with a structure designed to optimize the solubility and stability of drugs, has received much attention. Structurally, captisol is a modified β-CD macrocycle, i.e., a cyclic hydrophilic oligosaccharide, where four secondary alcoholic groups in the wider rim and three alternate primary alcoholic groups in the narrow rim of β-CD have been substituted by sulfobutylether chains (Scheme 1) (Jain et al., 2011; Shinde et al., 2015). Since the portals are elongated with S${\text{O}}_{3}^{-}$ associated sulfobutyl chains, in effect, the hydrophobicity of the cavity is extended on either side keeping the integrity of the core cavity of β-CD intact and the extended portal regions on either side perform as good cation receptors. The solubility of captisol in water is much higher than the native β-CD, has an advantageously low degree of toxicity and it does not show nephrotoxicity connected with β-CD (Stella and Rajewski, 1992; Jain et al., 2011). It also increases the solubility and stability of poorly water-soluble drugs (Fukuda et al., 2008; Kulkarni and Belgamwar, 2017). In our recent study, we have established the contrasting recognition behavior of parent β-cyclodextrin and captisol (SBE_7_βCD) toward a fluorescent probe, 4′,6-Diamidino-2-phenylindole (DAPI) and its utility toward stimuli-responsive on-off switches (Shinde et al., 2015). On its technological application, our recent work on ultra-bright rhodamines with SBE_7_βCD (captisol) in water has demonstrated the construction of a practical water-based dye laser system (Khurana et al., 2018). Further studies on its biological/medicinal application, we have established that the benign captisol macrocycle can be effectively used to inhibit and disintegrate amyloid fibrils/plaques, signifying its role as therapeutic agent toward neurodegenerative diseases, such as Alzheimer's and Parkinson's diseases (Shinde et al., 2017). In the present study, we anticipate that the interaction of negatively charged captisol portals with the four N-methylpyridyl ends of TMPyP will prevent the inherent self-assembling behavior of TMPyP and will deaggregate the π-stacked porphyrins, which will improve its photophysical properties and enhance their active singlet oxygen yield. Herein, we report the construction of supramolecular nanorods of 5,10,15,20-tetrakis(4-*N*-methylpyridyl)porphyrin dye/drug with captisol through hos-guest interaction and demonstrate its phototherapeutic application as effective photosensitizer and a superior antibacterial and antitumor agent.

## Materials and Methods

### Materials

Sulfobutylether-β-cyclodextrin sodium salt, commercially known as captisol, with a degree of substitution of 6.4 was obtained from Advent ChemBio Pvt. Ltd., India and used without further purification. The tosylate form of TMPyP obtained from Aldrich was converted to its chloride form by using an anion exchange resin (Mohanty et al., 2008). In the present study, the experimental solutions were prepared by using nanopure water obtained from a Millipore Elix 3/A10 water purification system (conductivity <0.1 μS cm^−1^).

### Spectroscopic and Imaging Methods

Absorption spectra measurements were carried out with a Jasco UV–Vis spectrophotometer (model V-650), and the concentration of TMPyP was calculated using the molar extinction coefficient of 2.26 × 10^5^ M^−1^ cm^−1^ at 422 nm (Mohanty et al., 2008). Steady-state fluorescence spectra were recorded using FS5 spectrofluorometer (Edinburgh Instruments). Dark toxicity, anti-tumor and antibacterial activity measurements were performed in PBS buffer, unless specified otherwise. Fluorescence quantum yield of the Captisol:TMPyP complex was measured by comparing the area under the curve with that of free TMPyP in water (Φ_f_ = 0.047) (Mohanty et al., 2008). Time-resolved fluorescence and anisotropy experiments were performed using a time-correlated single photon counting (TCSPC) spectrometer (Horiba Jobin Yvon, U.K.) and the particulars are provided in Supplementary Information. Dynamic light scattering (DLS) measurements were carried out using Malvern 4800 Autosizer.

Scanning Electron Microscope (SEM), Fluorescence Microscope (FM), and Atomic Force Microscopic (AFM) images of TMPyP and Captisol:TMPyP samples were recorded from drop casted samples on compatible solid substrates and the experimental details are given in Supplementary Information.

### Photostability Measurements

TMPyP and Captisol:TMPyP systems were irradiated with low irradiance light from a 150 W Xenon lamp (fluence rate ~80 μW cm^−2^ for 1 h at 422 ± 2.5 nm). The photodegradation of these systems was monitored by measuring the absorbance at different times at 422 nm.

### Singlet Oxygen (^1^O_2_) Generation Measurements

We have adopted the reaction of ^1^O_2_, generated from TMPyP or Captisol:TMPyP system on light irradiation, with 1,3-diphenylisobenzofuran (DPBF) to evaluate the quantum yield of ^1^O_2_ from these systems (Pradeepa et al., 2013). Two ml of air-saturated DPBF solution containing TMPyP or Captisol:TMPyP in DMF in a quartz cuvette was irradiated at 510 nm (to avoid the direct excitation of DPBF). The depletion of DPBF were followed by monitoring the decrease in optical density at 417 nm. Since TMPyP is having sufficient absorbance at ~417 nm, control experiments without DPBF were carried out to determine the actual decrease in the absorbance of DPBF. [Ru(bpy)_3_]^2+^ was used as reference and its ^1^${\text{O}}_{2}^{*}$ generation yield is considered as ~0.81 in air-saturated methanol (Abdel-Shafi et al., 2000).

### Antibacterial Activity Measurements

A single isolated colony of *Esherichia coli* from the Luria agar plate was transferred to 5.0 ml of Luria broth and incubated at 35 ± 2°C for 18–20 h. 5 μl of the culture was then transferred to fresh medium (5.0 ml LB) and incubated at 35 ± 2°C for 18–20 h. The culture was diluted to obtain 10^7^ cfu/ml using 0.85% saline. The cells were incubated in dark with TMPyP or Captisol:TMPyP systems for 15 min. (15.0 ml culture containing 100 μl of dye or complexed dye) and then exposed to white light (LED, fluence rate ~50 mW cm^−2^) at different time durations. Appropriate dilutions (10^4^, 10^3^) were spread plated (100 μl) on previously prepared Luria agar plates. The plates were incubated at 35 ± 2°C for 18–20 h and the colonies were counted. The plates unexposed to light served as control.

### Photosensitization Activity in Tumor Cells

MTT assay was carried out for the evaluation of cytotoxicity for TMPyP and Captisol:TMPyP systems in normal Chinese Hamster Ovary (CHO) cell line without white light irradiation and in human lung carcinoma A549 cell line with and without white light irradiation (LED, fluence rate ~50 mW cm^−2^). Both cell types were cultured as monolayers in phenol red free DMEM medium supplemented with 10% FBS, 100 μgmL^−1^ streptomycin and 100 Uml^−1^ penicillin at 37°C under 5% CO_2_ and humidified air. In brief ~2 × 10^4^ cells in 200 μl of phenol free DMEM medium in 96 well plate were treated with the desired concentrations of samples and incubated at 37°C for 2 h. Following this, cells were exposed to white light for 30 min, cultured for 48 h in the humidified incubator and processed for MTT assay as described previously (Mosmann, 1983). The bight field images of the cells were captured using Olympus fluorescence microscope (Model-CKX41, Japan) attached to a ProgRes® digital camera. For determining the dark toxicity, cells treated with TMPyP and Captisol:TMPyP systems were cultured for 48 h without exposing to white light and processed for MTT assay. The control group represents cells grown in DMEM medium without any treatment. The percentage of cell viability was calculated from the decrease in absorbance at 570 nm of treated groups as compared to that of control group. The experiment was done in triplicates (*n* = 3). The statistical significance of the variability among the means of treatment groups was determined by *T*-test and *P* < 0.05 considered significant.

## Results and Discussion

### Absorption and Emission Behavior of TMPyP With Captisol

**Figure 1:**
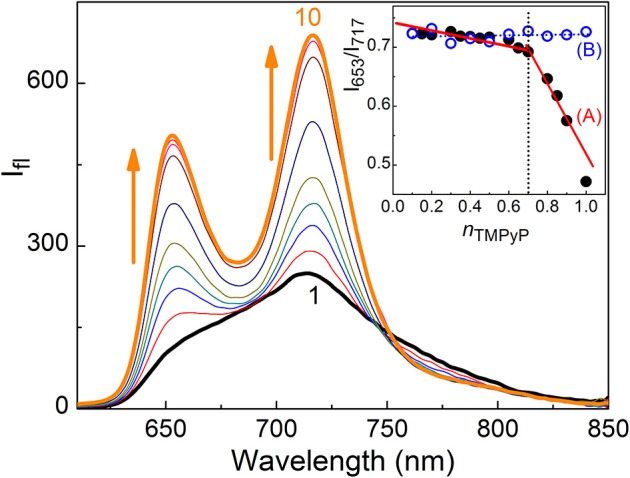
Fluorescence spectra of 2.1 μM TMPyP with captisol in H_2_O, λ_exc_ = 435 nm. [captisol]/μM: (1) 0.0, (2) 0.25, (3) 0.5, (4) 0.75, (5) 1.0, (6) 1.5, (7) 3.5, (8) 7.0, (9) 14.8, and (10) 22.0 at pH ~7.4. Inset shows Jobs plot using the fluorescence intensity ratio (*I*_717_/*I*_653_) with mole fraction of TMPyP, *n*_TMPyP_, for the Captisol:TMPyP complex **(A)**, where [captisol] + [TMPyP] = 10 μM, and only TMPyP solution **(B)** under identical concentration conditions.

TMPyP shows absorption in the wavelength range 350–700 nm including the soret and Q-bands (Mohanty et al., 2008). Initial addition of captisol upto ~1.5 μM to the aqueous solution of TMPyP results a decrease in the absorbance, further addition of captisol leads to increase in the absorbance along with small bathochromic shift ~4 nm in the soret band (Figure S1). The strong interaction between TMPyP and captisol is clearly visualized by the drastic change in the broad fluorescence band (due to intramolecular charge transfer between the *N-*methylpyridinium ring and the central porphyrin moiety of TMPyP) (Mohanty et al., 2008) and a reasonable enhancement in the fluorescence yield from 0.047 to 0.08 (Mohanty et al., 2008). The aggregation-induced/intramolecular charge transfer fluorescence self-quenching of TMPyP is largely suppressed in their aggregates by the bulky captisol host that are non-covalently attached on the porphyrin aromatic rings. The fluorescence band resolves into two narrow bands with maximum at 653 and 717 nm and a trough at 683 nm along with the variation in the fluorescence intensity ratio of 653–717 nm (I_653_/I_717_) with the increasing concentration of captisol (Figure 1). Very low concentration (3.5 μM) of captisol is sufficient to attain saturation, indicating strong binding interaction between captisol and TMPyP (Figure S2A).

**Figure 2:**
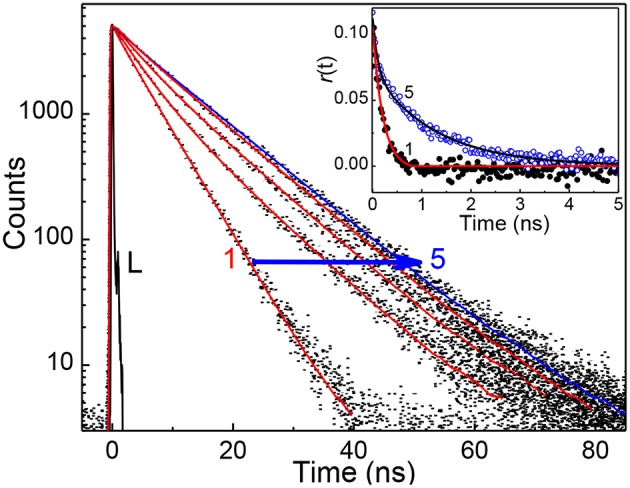
Fluorescence decay traces (λ_ex_ = 445 nm, λ_mon_ = 650 nm) of ~2 μM TMPyP solution at different concentration of captisol. [Captisol]/μM: 0.0 (1), 0.25 (2), 0.5 (3), 1.0 (4), and 22.0 (5). L represents the instrument response function. The inset displays the fluorescence anisotropy traces under the conditions for the fluorescence decay traces **1** and **5**.

In view of the modulations in the absorption and emission spectra and the high affinity of captisol toward N^+^-CH_3_, an inclusion of the pyridinium arm of TMPyP into captisol would be the most probable interaction mode. This type of encapsulation will interrupt the intramolecular charge transfer between the central porphyrin moiety and the *N-*methylpyridinium ring, which in turn disturbs the electronic distribution, bringing about two discrete emission bands centered at 653 and 717 nm, consistent with the Q(0,0) and Q(0,1) transitions (Vergeldt et al., 1995). These variations are markedly different from those observed on interaction of TMPyP with parent β-cyclodextrin (β-CD), where the *I*_653_/*I*_717_ ratio remains almost constant with observable variations in the trough region (Cosma et al., 2006). The influence of captisol binding on the excited state properties of TMPyP was clearly observable in the changes of its excited state lifetime as well. Figure 2 shows the fluorescence decay traces of TMPyP recorded in a TCSPC setup at 650/710 nm with increasing concentrations of captisol. The free TMPyP dye shows single exponential fitting with a lifetime value ~5.2 ns. Upon addition of increasing concentration of captisol to the TMPyP solution, the decay traces become biexponential. A long component appears at ~11 ns which is attributed to the lifetime of the complex. With increasing concentration of captisol, the relative amplitude of the free TMPyP decreases and the relative amplitude for the complex increases. The average lifetime increases from 5.2 to 11.0 ns. The list of fluorescence lifetimes with relative amplitudes is provided in Table S1. As claimed above, the strong complexation interaction at the *N-*methylpyridinium rings of TMPyP arrest the otherwise active intramolecular charge transfer pathways, thereby allowing extended lifetime for the excited singlet state, which may become advantageous for its photosensitizing and other photochemical features. Considering the availability of four such pyridinium moieties, TMPyP is expected to undergo multiple interactions with captisol, as observed with other hosts like calixarenes (Lang et al., 2001; Moschetto et al., 2002), cucurbiturils (Mohanty et al., 2008) and cyclodextrins (Moschetto et al., 2002; Cosma et al., 2006). In other words, the feasibility for such multiple binding would support for an extended/networked arrays of Captisol:TMPyP complex, realizing the formation of a new supramolecular assembly with altered photophysical properties of the TMPyP dye/drug.

To determine the binding stoichiometry, we monitored the ratio of the fluorescence intensities at 653 and 713 nm with varying mole fractions of TMPyP and captisol and the plots are shown in the inset of Figure 1. A distinct inflection in the slope close to 0.66 mole fraction of TMPyP (Figure 1A, inset trace), as compared to that obtained for the TMPyP alone in a similar experiment (Figure 1B, inset trace), proposes a 1:2 stoichiometry for the Captisol:TMPyP complex. The formation of a 1:2 complex is further recognized by the values obtained from time-resolved fluorescence anisotropy measurements. Due to complex formation there will be increase in the molecular hydrodynamic volume of the fluorophore, which will reflect in its rotational correlation time constant (τ_r_) evaluated from the anisotropy decay and the details are provided in the Supplementary Information (Method M1). The inset of Figure 2 displays the fluorescence anisotropy decay traces of TMPyP measured at 650 nm in the presence (trace 5) and absence (trace 1) of captisol. A single exponential decay analysis provided τ_r_ as 1.28 ns for the Captisol:TMPyP complex, against the 0.21 ns estimated for the free TMPyP. Since the radius of free TMPyP is ~9.7 Å (Mohanty et al., 2008), the above significant change in the τ_r_ value specifies that the radius of the emitting Captisol:TMPyP complex increases by about 8.0 Å as compared to TMPyP (Valeur, 2002; Lakowicz, 2006), suggestive of a complete inclusion of the pyridyl arm into the extended cavity of captisol with the positively charged nitrogen closer to the sulfonate groups. Such an arrangement is in good support for the assertion of 1:2 (Captisol:TMPyP) complex formation as envisaged in Scheme 1.

### ^1^H NMR Measurements

The formation of host-guest complexation is further supported by the changes in the chemical shift observed on the α- and β-pyridyl protons as well as the >NC${\text{H}}_{3}^{+}$ protons of TMPyP in the presence of captisol (Figure S3). These three different protons of TMPyP show large downfield shift in the ^1^H NMR signal ranging from 0.027 to 0.171 in the presence of 1 equivalent of captisol with respect to TMPyP. These positions were further shifted slightly by increasing the host concentration to 2 equivalents of captisol. This result points to the deshielding of the electron distribution in the N-methylpyridyl rings reside near to the sulfonate groups of captisol through strong electrostatic interactions. It may be noted that in the present case, separate signals for bound and unbound N-methylpyridyl protons were not observed which may be due to a probable faster host-guest exchange process in the NMR time scale that leads to the observation of N-methylpyridyl proton resonance at an average position.

### Isothermal Titration Calorimetric Measurement

**Figure 3:**
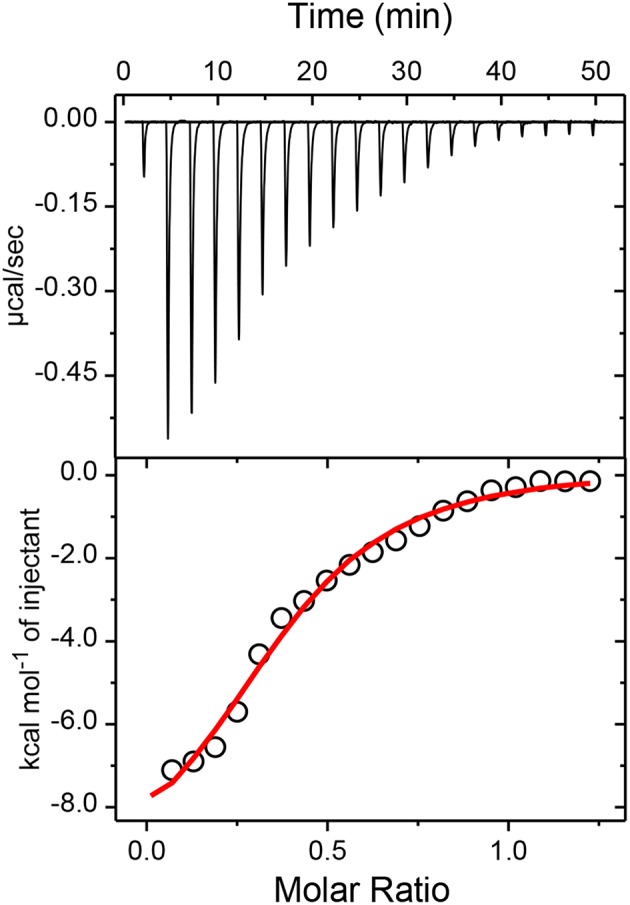
Upper panel shows the raw data for the titration of 100μM TMPyP with 600μM captisol at pH 7.4 in phosphate buffer (10 mM) and 25°C, showing the calorimetric response as successive injections of the host are added to the sample cell. Lower panel shows the integrated heat profile of the calorimetric titration given in the upper panel. The solid line represents the best non-linear least-squares fit to a sequential binding- site model. The changes in the enthalpy and entropy values and the binding constant values at each step complexation are presented in the lower panel.

To confirm the proposed 1:2 stoichiometry of the host-guest complex, isothermal titration calorimetric (ITC) measurements have been carried out considering captisol as host and TMPyP as guest/ligand. The integrated heat profile *vs*. the mole ratio has been generated from the heat evolved during the titration and is presented in Figure 3, which gave a satisfactory fit for a sequential 1:2 binding model. The overall binding constant value [*K* = *K*_1_× *K*_2_ = (3.0 × 10^4^ M^−1^) × (3.1 × 10^4^ M^−1^)] was estimated as 9.3 × 10^8^ M^−2^. The negative enthalpy changes (ΔH_1_ = −2.7 kcal mol^−1^ and ΔH_2_ = −7.5 kcal mol^−1^) during the two step complexation processes indicate that the binding interactions are thermodynamically favorable. Moreover, a higher negative enthalpy in the second binding step suggests a cooperative mechanism for higher order self-assembly process of TMPyP and captisol in aqueous medium. The estimated negative ΔG values from enthalpy and entropy changes indicate the feasibility of the complex formation. The detailed binding constant values along with thermodynamic parameters obtained from the ITC plots are provided in Note S1. We would like to state here that the ITC data also fits to a sequential 1:4 binding model considering captisol as ligand which point toward the formation of extended structures.

### DLS Measurements

To explore the formation of extended assembly from Captisol:TMPyP complex leading to larger assembly/particles, we carried out DLS measurement of TMPyP at different concentrations of captisol. From the size distribution curve obtained on addition of captisol to TMPyP solution (Figure S4), the particle size has been evaluated which increased gradually from 143 to 190 nm, 900 and 1,650 nm, respectively with 250, 500, 1,000, and 2,000 μM of captisol (Table S2). This confirms the formation of extended assembly/large moieties and is quite reasonable by virtue of the multiple binding sites available on both the host and the guest.

### SEM and FM Measurements

**Figure 4:**
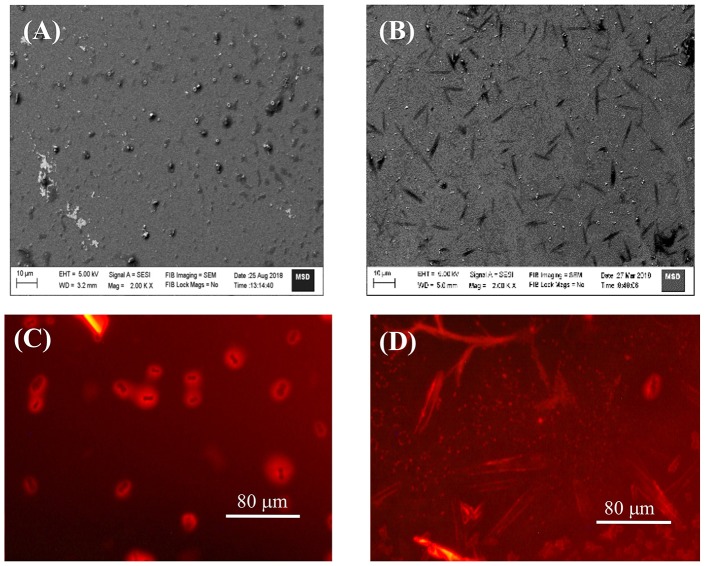
SEM **(A,B)** and FM **(C,D)** images of TMPyP alone **(A,C)** and TMPyP (2 μM) with captisol (25 μM) **(B,D)** using green light excitation.

As envisaged, the strong and multiple binding eventually lead to extended/self-assembled supramolecular structures. In this experiments we have employed SEM and FM method to look in to such probable nanostructures in the samples drop casted on silica wafer/cover slip, by monitoring the surface morphology of TMPyP in the absence and presence of captisol (Figures 4A,B and Figures S5A1–A4). We have added excess concentration of captisol to the TMPyP solution to have >95% complex formation in the solution. SEM images recorded from the sample of Captisol:TMPyP complex displayed distinctive nanorods of ~10 μm length, whereas free TMPyP under similar conditions displayed only lumps of aggregated dyes without any discrete morphology. The confirmation that these nanorods do incorporate the TMPyP chromophore came from the fluorescence microscopy (FM) images (Figures 4C,D) obtained from the free and captisol complexed TMPyP samples casted on pre-cleaned glass surface. Bright orange fluorescent streaks of micron length structures seen from images asserts that the captisol complexed TMPyP do grow in extended supramolecular structures, whereas the TMPyP alone remains as aggregated lumps of no specific morphology. The smaller nanorods (~2 μm) of Captisol:TMPyP complex are also seen in the AFM images (Figures S5B1,B2).

### Photostability and Singlet Oxygen Generation Measurements

**Figure 5:**
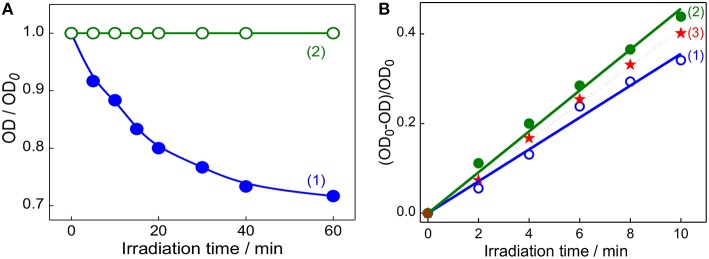
**(A)** Changes in the absorbance of TMPyP monitored at 422 nm on irradiation at 422 ± 2.5 nm using 150 W Xenon lamp from the steady state fluorimeter; TMPyP alone (1) and Captisol:TMPyP complex (2). **(B)** The consumption of DPBF as a function of irradiation time in the air-equilibrated DMF solution of DPBF and TMPyP (~2 μM) in the absence (1), presence of 20 μM captisol (2). Trace (3) represent the singlet oxygen yield evaluated for [Ru(bpy)_3_]^2+^ as standard (Φ(^1^${\text{O}}_{2}^{*}$) = 0.81) in air equilibrated CH_3_OH) under similar irradiation conditions.

After establishing the formation of captisol assisted supramolecular nanorod assembly of TMPyP with improved excited state features, we were inquisitive to investigate its photostability and the ability to generate singlet oxygen (^1^${\text{O}}_{2}^{*}$) which are essential to assess their practical use, particularly as photosensitizer in photodynamic therapy. In most of the dye-based PDT systems, the unspecific surface adsorption, aggregation propensity and photobleaching of the chromophore reduce their photosensitizing efficacy. However, such unspecific aggregation interactions and non-radiative pathways are largely prevented on macrocyclic encapsulation of the dye/drug. Apparently, in the host confined environment, the excited singlet state of the dye/drug find relatively longer lifetime favoring more triplet yield (Bhasikuttan et al., 2011). To take advantage of the improved photophysical features of Captisol:TMPyP complex and to attest its improved photostability, we have irradiated the aqueous solution of TMPyP in the absence and presence of captisol host using low irradiance light from a 150 W Xenon lamp (fluence rate ~80 μW cm^−2^ for 1 h at 422 ± 2.5 nm). From the corresponding absorption spectra (Figure S6) and the plot of the changes in the absorbance at the Soret band position (422 nm) with irradiation time monitored both in the presence and absence of captisol (Figure 5A), displayed remarkable improvement in the photostability of TMPyP when it is complexed with captisol.

On the other hand, generation of reactive singlet oxygen is considered to be the central issue of PDT procedure (Mehraban and Freeman, 2015). In this regard, porphyrins and expanded porphyrins are under intense investigation due to their photosensitizing ability for PDT application (Kou et al., 2017). These photosensitizers are activated on exposure to light and become photosensitizers' triplet, which react with dissolved molecular oxygen to produce the reactive singlet oxygen (^1^${\text{O}}_{2}^{*}$), which are responsible for therapeutic action (Mehraban and Freeman, 2015; Kou et al., 2017). On the basis of our present finding that the Captisol:TMPyP complex displayed enhanced excited singlet state lifetime and photostability, we foresee that this supramolecular assembly of TMPyP can largely enhance the singlet oxygen generation favoring PDT. Following the established method of measurement, we have adopted the reaction of singlet oxygen with 1,3-diphenylisobenzofuran (DPBF) and the consumption of DPBF as a measure of the yield of singlet oxygen generation in the system (Pradeepa et al., 2013). Due to poor solubility of DPBF in water, these measurements were carried out in DMF solvent. We have verified that the complexation interaction of TMPyP with captisol follows comparable binding behavior in DMF and gets saturated at slightly higher concentration of captisol as compared to the captisol concentration in H_2_O medium (Figure S2B).

Figure 5B shows the changes in the absorbance of DPBF monitored at 425 nm upon photoirradiation of air-saturated DMF solution at 510 nm (which is selected to avoid the direct excitation of DPBF) containing DPBF and TMPyP in the presence/absence of captisol. Similar irradiation studies were also carried out with [Ru(bpy)_3_]^2+^system as control to correlate with the change in the singlet oxygen generation with time. As displayed in trace 1 and 2 (Figure 5B), the yield of singlet oxygen increases linearly with irradiation time and the photogenerated singlet oxygen quantum yields from TMPyP and its captisol complex were estimated to be 0.73 and 0.95, respectively, in comparison with that of reference ([Ru(bpy)_3_]^2+^, Φ(^1^${\text{O}}_{2}^{*}$) = 0.81 in air-saturated methanol (trace 3)) (Method M2) (Abdel-Shafi et al., 2000). The significant enhancement (0.73–0.95) in the efficiency to generate singlet oxygen and the photostability of TMPyP in the presence of captisol is attributed to the enhancement of excited state lifetime and the concurrent improvement in the triplet yield. Due to the overlapping absorption spectra of DPBF and TMPyP, the control experiments have been performed in the absence of DPBF to take care of the absorption loss due to TMPyP and the complex during photoirradiation. To document the constructive role of the captisol host, it may be stated here that similar studies on TMPyP in presence of parent β-CD host, having no extended sulfobutyl arms, provided singlet oxygen yield of 0.47, which is much less than that observed for free TMPyP itself.

### Photosensitized Antibacterial and Antitumor Activities

**Figure 6:**
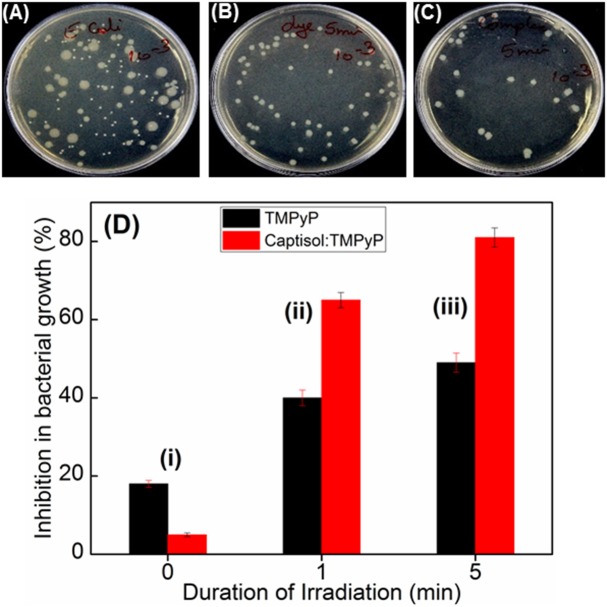
Images of plates showing bacterial growth of *E. coli* in terms of colonies in the absence of any additive **(A)** and presence of TMPyP (5 μM) **(B)** and with TMPyP(5 μM):captisol (20 μM) **(C)** at pH 7.5 after white light irradiation for 5 min. **(D)** is the bar chart representation of the percentage of inhibition in bacterial growth byTMPyP (5 μM) in the absence (black bar) and presence (red bar) of captisol (20 μM) with irradiation time 0 min (i); 1 min (ii); 5 min (iii) against *E. coli* (Gram –ve) bacteria.

Quaternary ammonium compounds are broadly used as antibacterial agents to kill various types of bacteria (Zhu et al., 2011; Chen et al., 2017). Since methylpyridinium moiety in TMPyP contains quaternary ammonium group, we were interested to investigate whether the enhanced singlet oxygen yield of TMPyP in the presence of captisol will have any effect on the antibacterial activity. In this perspective, we have investigated the antibacterial activity of TMPyP and the Captisol:TMPyP complex with and without light irradiation against a pathogenic Gram-negative micro-organism i.e., *Escherichia coli* (*E. coli*) and Gram-positive micro-organism *Staphylococcus aureus*, by well-known spread plating method (Figures 6A–C and Figure S7). It is observed that in the unirradiated condition (Figure 6Di) there is a nominal inhibition (~18%) in the bacterial growth in the presence of TMPyP and the inhibition is reduced to ~5% in presence of Captisol:TMPyP complex, indicating that the Captisol:TMPyP complex is less toxic under dark conditions. However, assessment of the bacterial growth from the plates containing TMPyP or Captisol:TMPyP complex and irradiated with white light (LED, fluence rate ~50 mW cm^−2^) showed notable inhibition in the growth of colonies of *E. coli* as compared to their respective blanks having no TMPyP or the complex (Figures 6A–C). More importantly, the extent of inhibition (or the antibacterial activity) is found to be significantly higher in the irradiated plates containing the Captisol:TMPyP complex and are found to be effective in killing ~81% of the bacteria on ~5 min white light irradiation, as compared to ~49% by TMPyP alone under similar conditions (Figure 6Diii). Comparable killing effect was also observed with Gram positive *Staphylococcus aureus* bacteria in a similar experimental follow up (Figure S7). This enhanced antibacterial activity in the Captisol:TMPyP complex is in good agreement to the contention of captisol assisted enhancement in the generation of reactive singlet oxygen by TMPyP dye/drug, mainly due to its modulation in the excited state dynamics.

**Figure 7:**
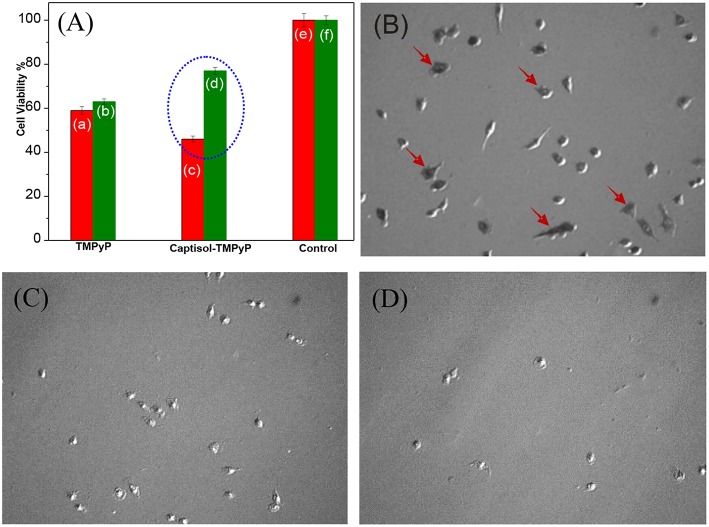
**(A)** Cell viability studies carried out in lung carcinoma A549 cell lines using MTT assay with the addition of respective TMPyP, Captisol:TMPyP and Control systems under white light irradiation (red bars, **a,c,e**) and in dark conditions (green bars **b,d,f**). The circled portion indicates the enhanced toxicity of Captisol:TMPyP complex in the A549 cancer cell lines under white light irradiation (For comparison with normal CHO cell lines see Figure S8). **(B–D)** The phase contrast images of living lung carcinoma A549 cells in DMEM medium in the absence of any additive treated as control **(B)**, in the presence of TMPyP (5 μM) **(C)** and in the presence of Captisol (75 μM):TMPyP (5 μM) complex **(D)**, after 30 min white light irradiation during MTT assay. The arrow indicates presence of formazan crystal.

Studies have been further extended to explore the photosensitizing effect of the Captisol:TMPyP complex toward cancer cells on white light irradiation (LED, fluence rate ~50 mW cm^−2^) using MTT assay. Though the imaged assemblies/nanorods are of micron size, quite large for cellular uptake, it may be noted here that the basic unit of the assembly is of only few nanometer size and beside the micron size extended assemblies, there are smaller assemblies/particles of ~100–150 nm of size in solution, which can contribute to the cellular effect. Figure 7A shows the percent viability of A549 cells (human lung carcinoma) under different treatment conditions. It clearly indicated that treatment with TMPyP alone without white light irradiation (Figure 7Ab) led to significant decrease (*P* < 0.05) in viability of A549 cells suggesting its cytotoxic effect. As expected the exposure to white light further enhanced the cytotoxic effect of TMPyP (Figure 7Aa). Notably the cytotoxic effect of Captisol:TMPyP system on white light irradiation (Figures 7A,C) was significantly (*P* < 0.05) higher as compared to that of TMPyP (Figure 7Aa). This confirmed the improvement in photosensitizing effect of TMPyP upon complexation with Captisol. These results were also supported by the bright field images showing reduction in the number as well as change in morphology of A549 cells treated with TMPyP or Captisol:TMPyP under white light irradiation as compared to control cells (Figures 7B–D). The morphology of a cell is an indicative of its viability or health. The control cells presented in Figure 7B show a very distinct polymorphic morphology. Additionally, this group also shows the formation of purple formazan crystal within cells which confirms their viability. On contrary, cells treated with TMPyP or Captisol:TMPyP exhibited reduction in size and number, change in morphology (rounding) and minimal formation of purple formazan crystal. All these changes are attributed to the cytotoxicity induced by TMPyP or Captisol:TMPyP. Interestingly, as indicated in Figures 7Ab,d, it was seen that under the dark condition the cytotoxic effect of Captisol:TMPyP was significantly (*P* < 0.05) lower as compared to that of TMPyP alone in A549 cells. To further validate this result, the dark toxicity of TMPyP and Captisol:TMPyP systems was also evaluated in normal epithelial (CHO) cells and the results were found to be in similar lines (Figure S8). Moreover, the cell viability data obtained with the captisol alone revealed no cytotoxicity with ~75 μM of captisol, which is used in these studies. Similar non-toxic data were observed in our earlier study using insulin fibrils (Shinde et al., 2017), where we did not observe any cytotoxicity effect of captisol up to ~250 μM using CHO cell lines. Thus, it is confirmed that complexation of TMPyP with Captisol reduced its inherent toxicity. Above observations gain a lot of significance as it has profound advantage in reducing the normal cells toxicity of TMPyP and improving its singlet oxygen generation ability and thereby enhancing its antitumor activity/photodynamic therapy.

## Conclusion

In summary, we have demonstrated the construction of a supramolecular assembly of TMPyPdye/drug with captisol macrocycle through host-guest interaction. The presence of several binding sites both on TMPyP as well as on captisol allows strong multiple host-guest interactions, building an extended supramolecular assembly containing TMPyP and is imaged as nanorods of ~10 μm in length or more. Detailed spectroscopic and imaging measurements revealed that the Captisol:TMPyP assembly exhibits a number of advantageous features over the uncomplexed TMPyP dye, namely, increased singlet state yield and lifetime, enhanced singlet oxygen yield, improved photostability, and overall, much better photosensitizing effect. Utilizing these features in its biological stride, enhanced antibacterial activity toward *E. coli* and increased cytotoxicity toward lung carcinoma A549 cells on light illumination and reduced dark toxicity toward normal cells have been demonstrated. All these synergistic effects of supramolecular nanorod formation of Captisol:TMPyP complex are beneficial for improving the efficacy of photodynamic therapy for the treatment of cancer as well as proves it to be a good antibacterial agent.

## Data Availability

The raw data supporting the conclusions of this manuscript will be made available by the authors, without undue reservation, to any qualified researcher. Requests to access the datasets should be directed to jyotim@barc.gov.in.

## Author Contributions

AB and JM conceived the project and designed the research work. Photophysical studies, singlet oxygen generation, and photostability studies were carried out by RK under the guidance of NB and JM. RK and ASK carried out the antibacterial studies with and without light irradiation under the guidance of SC. RK carried out the MTT assay with and without light irradiation under the guidance of AK. The manuscript was prepared by JM and edited by AK, JM, and AB.

### Conflict of Interest Statement

The authors declare that the research was conducted in the absence of any commercial or financial relationships that could be construed as a potential conflict of interest.
