# Supplementary material for: Supramolecular Nanorods of (N-Methylpyridyl) Porphyrin With Captisol: Effective Photosensitizer for Anti-bacterial and Anti-tumor Activities
Source: Front Chem. 2019 Jun 21;7:452. doi: 10.3389/fchem.2019.00452 (PMC6598724; doi:10.3389/fchem.2019.00452)
Supplement: Supplementary file 1 [file Data_Sheet_1.pdf]

Supplementary Material

Supramolecular Nanorods of (N-methylpyridyl) Porphyrin with Captisol: Effective Photosensitizer for Anti-bacterial and Anti-tumor Activities

Raman Khurana[1,2], Aarti S. Kakatkar[3], Suchandra Chatterjee[3], Nilotpal Barooah[1], Amit Kunwar[1,2], Achikanath C. Bhasikuttan[1,2]*, Jyotirmayee Mohanty[1,2]*

[1]Radiation & Photochemistry Division, Bhabha Atomic Research Centre, Trombay, Mumbai 400085, INDIA

[2]Homi Bhabha National Institute, Training School Complex, Anushaktinagar, Mumbai, 400094, INDIA

[3]Food Technology Division, Bhabha Atomic Research Centre, Trombay, Mumbai 400085, INDIA

1. Methods

1.1 Time-resolved fluorescence and anisotropy measurements using TCSPC

The time-resolved fluorescence measurements were carried out using a time-correlated single photon counting (TCSPC) set-up from Horiba Scientific (UK). In the present work, 445 nm (~100 ps, 1 MHz repetition rate) diode laser was used for excitation. A reconvolution procedure was used to analyze the observed decays (Lakowicz, 2006; O'Connor, and Phillips, 1984), which could be satisfactorily fitted by mono- or biexponential decay functions. A deconvolution procedure was used to analyze decays using a proper instrument response function obtained by using a light scatterer (suspension of TiO_2 particles in water). The fluorescence decays [I(t)] were analyzed in general as a sum of exponentials (Lakowicz, 2006; O'Connor, and Phillips, 1984),

$$I(t) = \sum B_i \exp(-t / \tau_i) \qquad \text{(S1)}$$

where, B_i and τ_i are the pre-exponential factor and fluorescence lifetime for the ith component, respectively. Reduced chi-square (χ^2) values and random distribution of the weighted residuals among data channels were used to judge the acceptance of the fits.

For anisotropy measurements, samples were excited with a vertically polarized excitation beam and the vertically and horizontally polarized fluorescence decays were collected with a large spectral bandwidth of ~32 nm. Using these polarized fluorescence decays, the anisotropy decay function, *r(t)*, was constructed as follows (Lakowicz, 2006; O'Connor, and Phillips, 1984):

$$r(\mathrm{t}) = \frac{I_{\mathrm{V}}(\mathrm{t}) - G I_{\mathrm{H}}(\mathrm{t})}{I_{\mathrm{V}}(\mathrm{t}) + 2G I_{\mathrm{H}}(\mathrm{t})} \quad \text{(S2)}$$

I_V(t) and I_H(t) are the vertically and horizontally polarized decays, respectively, and G is the correction factor for the polarization bias of the detection setup. The G factor was determined independently by using a horizontally polarized excitation beam and measuring the two perpendicularly polarized fluorescence decays. ^{1}H NMR spectra were recorded on a Bruker Avance WB 600 MHz spectrometer at TIFR, India.

1.2 DLS Measurements

The dynamic light scattering (DLS) measurements were carried out using Malvern 4800 Autosizer employing an Ar ion laser (λ=514.5 nm) and digital correlator. The scattered light intensity was monitored at a scattering angle of 90 and the intensity correlation function over a time range of 10^{-6} to 1s was computed.

1.3 FM Measurements

Fluorescence microscopy (FM) images were obtained from Olympus fluorescence microscope (Model – BX53, Japan) attached to Progres® digital camera. The samples for FM images were drop casted on a pre-cleaned coverslip and left for drying for few hours. All the experimental measurements were performed at ~25 °C.

1.4 AFM Measurements

The recording of Atomic Force Microscope (AFM) images was carried out using a NT-MDT solver model P47 instrument (Russia) with 50 μm scanner head and silicon nitride tip in semi-contact mode. The sample for AFM measurement was prepared by drop casting a dilute solution on a mica sheet, followed by drying. AFM examinations were performed in ambient air with a commercial microscope.

1.5 SEM Measurements

Scanning Electron Microscope (SEM) analysis was carried out using field emission SEM (Zeiss Sigma FESEM 300). For this purpose, a drop of sample (~10^{-5} M solution) was deposited on a Si-wafer mounted on an Aluminum stub with the help of a double-sided adhesive tape. The samples were dried at room temperature and vacuum dried for 30 min to ensure complete removal of any residual water and coated with gold before being analysed.

2 Supplementary Data

2.1 Method M1

Theoretically, τ_r for the emitting species can be correlated to the viscosity (η) of the medium and its rotational diffusion coefficient (D_r) and is provided by the Stokes–Einstein relationship (eq. S3) (Lakowicz, 2006; O'Connor, and Phillips, 1984):

$$\tau_r = \frac{1}{6D_r} \quad \text{where} \quad D_r = \frac{\mathrm{RT}}{6\mathrm{V}\eta} \quad \text{(S3)}$$

Here, V is the hydrodynamic molecular volume of the complex, η is the viscosity of the medium and T is the absolute temperature.

2.2 Method M2

The quantum yield of singlet oxygen $\Phi(^1O_2^*)$ of the sample is calculated by a relative method comparing the quantum yield of $[Ru(bpy)_3]^{2+}$ photosensitizer ($\Phi(^1O_2^*)$ = ~0.81 in air-saturated methanol) as the reference. $\Phi(^1O_2^*)$ is calculated according to the following modified equation (Kochevar and Redmond, 1992).

$$\Phi(^1O_2^*)_S = \Phi(^1O_2^*)_R \times \frac{Slope_S}{Slope_R} \times \frac{OD_R}{OD_S} \times \frac{(\eta_S)^2}{(\eta_R)^2} \quad \text{(S4)}$$

Where, $\Phi(^1O_2^*)_S$ and $\Phi(^1O_2^*)_R$ are the singlet oxygen quantum yield of sample (S) and reference (R). 'Slope' is the slope of plot $((OD_0\text{-}OD)/OD_0)$ at 425 nm *versus* irradiation time (Fig. 5B). OD_S and OD_R are the respective OD of sample and reference solutions at the irradiation wavelength i.e. 510 nm. The OD at 510 nm is kept nearly the same for the TMPyP (sample) and the $Ru(bpy)_3^{2+}$ (reference) solutions. On irradiation, changes in the OD at 425 nm in the solution of DPBF without TMPyP if any, is corrected while calculating the actual changes at 425 nm in the presence of TMPyP. η_S and η_R are the refractive indices of the sample solution and reference solution, respectively. $(\eta_S)^2/(\eta_R)^2$ is the solvent correction factor which takes care of the experiments carried out in two different solvent media.

2.3 Note S1

From the ITC measurements the binding constants, enthalpy, entropy and free energy changes for the 1:2 captisol:TMPyP complex were evaluated and the values are:

$K_1 = (3.0\pm1.0) \cdot 10^4\ M^{-1}$, ΔH_1 = -2.7 kcal mol^{-1}, $-T\Delta S_1$ = -3.4 kcal mol^{-1}, ΔG_1 = -6.1 kcal mol^{-1} and $K_2 = (3.1\pm1.8) \times 10^4\ M^{-1}$, ΔH_2 = -7.5 kcal mol^{-1}, $-T\Delta S_2$ = 1.4 kcal mol^{-1}, ΔG_2 = -6.1 kcal mol^{-1} for captisol:TMPyP complexes. The binding of both the portals of captisol towards TMPyP results similar binding constant values. Hence, the overall binding constant (K) = ($K_1 \times K_2$) = $9.3 \times 10^8\ M^{-2}$.

3 Supplementary Figures and Table

3.1 Supplementary Figures

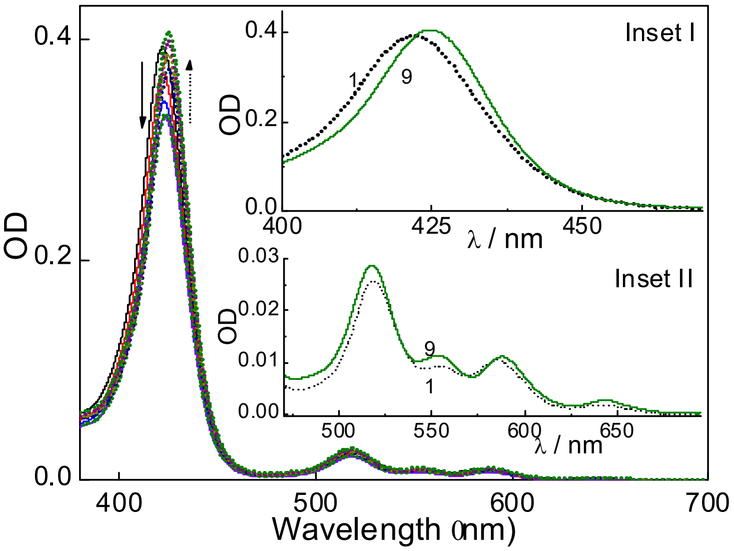

Figure S1. Absorption spectra (soret band) of TMPyP (2.1 μM) in aqueous solution at different concentrations of captisol. [Captisol]/μM: (1) 0, (2) 0.25, (3) 0.5, (4) 0.75, (5) 1.0, (6) 3.5, (8) 7.0, (8) 14.8 and (9) 22. Inset I and Inset II show the changes in the soret band and Q band (500-700 nm) region, respectively, in the presence of 22 μM captisol.

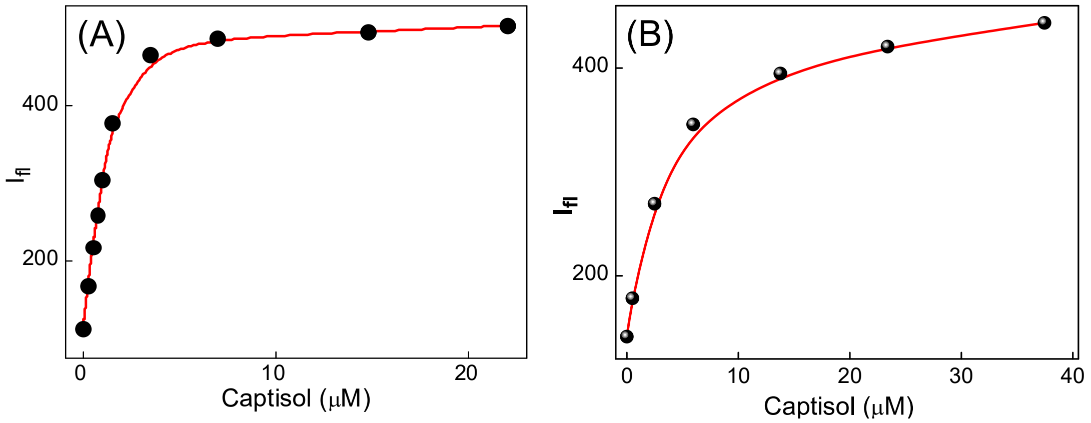

Figure S2. Plot of the changes in the fluorescence intensity (I_{fl}) with increasing concentration of Captisol at 660 nm in aqueous solution (A) and in DMF (B).

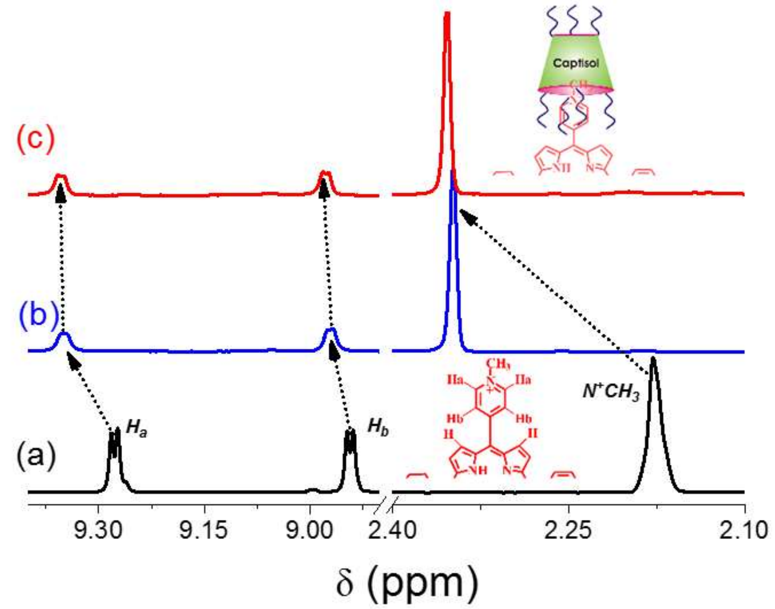

Figure S3. ^{1}H NMR spectra of (**a**) TMPyP, (**b**) TMPyP and captisol (1.0 equiv.) and (**c**) TMPyP and captisol (2.0 equiv.) in D_2O. Inset: N-methyl pyridyl moiety with a part of the central porphyrin moiety and the pictorial representation of the host interaction.

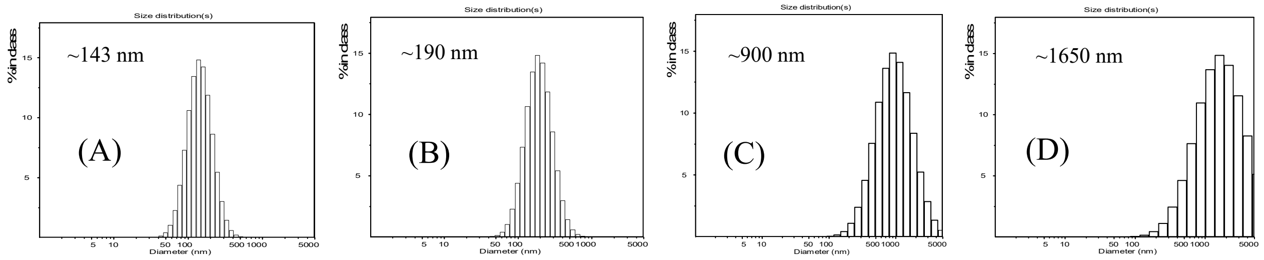

Figure S4. Size distribution curve obtained during addition of captisol to TMPyP solution indicating the formation of extended assemblies/moities.

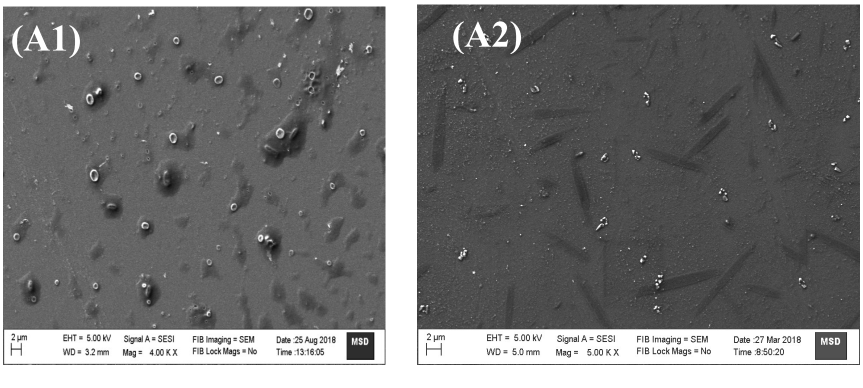

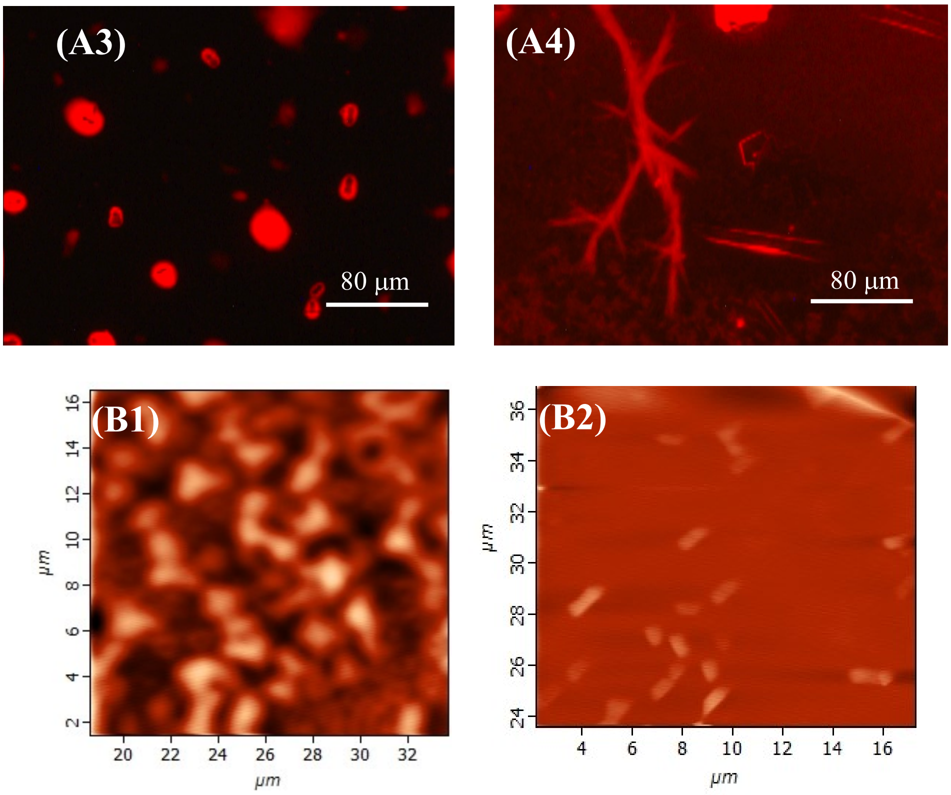

Figure S5. Additional images from SEM (A1, A2), FM (A3, A4) and AFM (B1,B2) measurements of (1) TMPyP and (2) TMPyP (2 μM) with captisol (25 μM).

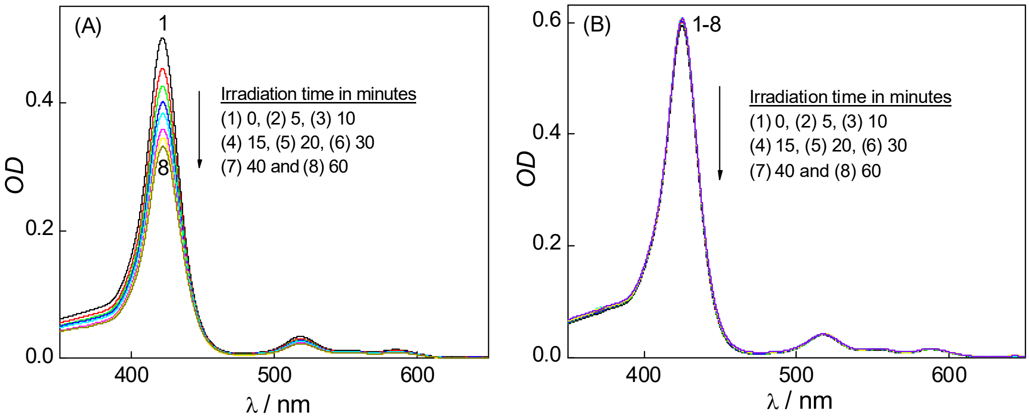

Figure S6. Photodegradation of TMPyP (ca. 2 μm) in aerated water in the absence (A) and presence (B) of 25 μm of captisol followed through the decrease of the visible absorption with increasing time of irradiation at 422 nm using a 150 W xenon lamp from a steady state fluorimeter.

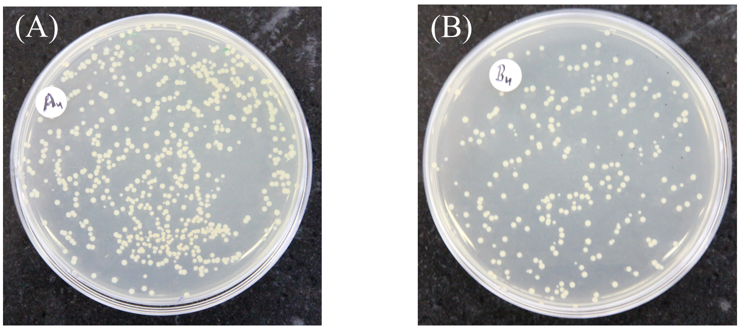

Figure S7. Plates showing growth of Gram positive *Staphylococcus aureus* in colonies with TMPyP (A) and Captisol:TMPyP complex (B) on light irradiation.

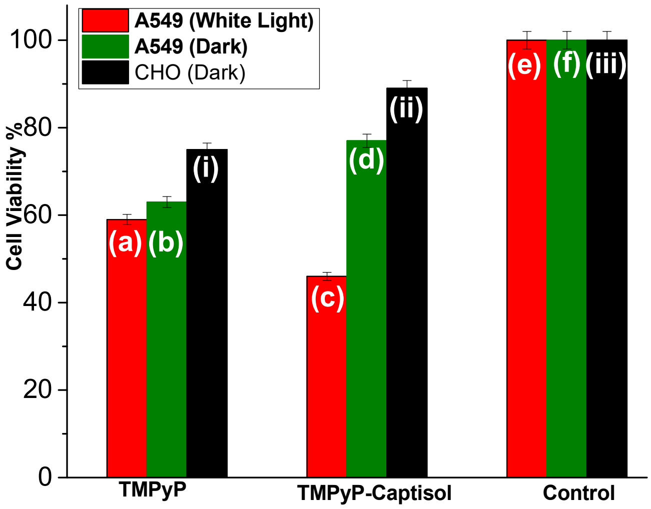

Figure S8. Cell viability studies carried out in lung carcinoma A549 cell lines (red and green bars) using MTT assay with the addition of respective TMPyP, Captisol:TMPyP and Control systems under white light irradiation (red bars, a, c, e) and in dark conditions (green bars b, d, f). The black bars (i, ii, iii) represent similar studies carried out in normal CHO cells under dark condition.

3.2 Supplementary Table

Table S1. Excited state decay time constants evaluated for captisol:TMPyP complex at varying concentration of captisol.

[Captisol] (μM)	τ_1 (ns)[#]	a_1 (%)	τ_2 (ns)	a_2 (%)	<τ> (ns)
0.0	5.2	100	---	---	5.2
0.25	5.2	52	11.0	48	8.0
0.5	5.2	25	10.8	75	9.4
0.75	5.2	13	10.9	87	10.2
1.0	5.2	8	11.0	92	10.5
1.5	5.2	7	11.1	93	10.7
3.5	5.2	5	11.2	95	10.9
15.0	5.2	4	11.2	96	11.0
22.0	5.2	4	11.2	96	11.0

Table S2: DLS measrements carried out in solutions of 500μM TMPyP with the addition of varying concentration of Captisol

	**[Captisol] (μM)**	**Paricle Size (nm)**
**A**	250	143
**B**	500	190
**C**	1000	900
**D**	2000	1650

References

Kochevar, I. E., and Redmond, R. W. (1992). Photosensitized production of singlet oxygen. *Methods Enzymol.* 319, 20-28.

Lakowicz, J. R. (2006). *Principles of Fluorescence Spectroscopy*. 3rd ed.; Springer: New York.

O'Connor, D. V., and Phillips, D. (1984). *Time correlated single photon counting*. Academic Press: New York.
